# Intact perception but abnormal orientation towards face-like objects in young children with ASD

**DOI:** 10.1038/srep22119

**Published:** 2016-02-25

**Authors:** Quentin Guillon, Bernadette Rogé, Mohammad H. Afzali, Sophie Baduel, Jeanne Kruck, Nouchine Hadjikhani

**Affiliations:** 1 URI Octogone, University of Toulouse, 5 Allée Antonio Machado, 31058 Toulouse Cedex 9, France; 2 Institut Universitaire de France (IUF) 1, Rue Descartes, 75231 Paris Cedex 05, France; 3 Harvard Medical School/MGH/MIT, Martinos Center for Biomedical Imaging, Building 75, 3rd Avenue, Charlestown, MA 02129, USA; 4 Gillberg Neuropsychiatry Center, University of Gothenburg, Sweden

## Abstract

There is ample behavioral evidence of diminished orientation towards faces as well as the presence of face perception impairments in autism spectrum disorder (ASD), but the underlying mechanisms of these deficits are still unclear. We used face-like object stimuli that have been shown to evoke pareidolia in typically developing (TD) individuals to test the effect of a global face-like configuration on orientation and perceptual processes in young children with ASD and age-matched TD controls. We show that TD children were more likely to look first towards upright face-like objects than children with ASD, showing that a global face-like configuration elicit a stronger orientation bias in TD children as compared to children with ASD. However, once they were looking at the stimuli, both groups spent more time exploring the upright face-like object, suggesting that they both perceived it as a face. Our results are in agreement with abnormal social orienting in ASD, possibly due to an abnormal tuning of the subcortical pathway, leading to poor orienting and attention towards faces. Our results also indicate that young children with ASD can perceive a generic face holistically, such as face-like objects, further demonstrating holistic processing of faces in ASD.

Face detection is an automatic, rapid and subconscious process, considered as a core component of the social perceptual system subtending social behaviors^1^. Faces can even be perceived in non-face stimuli, such as grilled toasts, clouds or landscapes (i.e. an illusory detection termed pareidolia)^2^. Detecting faces in non-face stimuli may have strong adaptive values. From an evolutionary point of view, the cost of erroneously detect a face in non-face stimuli might indeed be smaller than the one associated with failing to detect other’s face in the environment^3^. Furthermore, many studies demonstrated that newborns look longer and orient more frequently towards faces and face-like patterns rather than stimuli of similar complexity, suggesting a predisposition to detect, orient and attend to visual stimuli that most likely display a face^4^,^5^,^6^.

In the present study, we asked whether young children with Autism Spectrum Disorder (ASD) could orient to and perceive a face in non-face stimuli on the basis of a global face-like configuration (i.e. two eyes, above a nose which is above a mouth, also termed ‘first-order relational information’, see^7^).

Famous examples of non-face stimuli that can be perceived as face-like (referred to hereafter as ‘pareidolic faces’) are Mooney and Arcimboldo images^8^,^9^,^10^,^11^,^12^,^13^,^14^,^15^,^16^,^17^. Mooney and Arcimboldo images do not contain elementary facial parts but can still be perceived as a face due to the global face-like configuration formed by their parts. However, when presented upside down, pareidolic faces are no longer perceived as faces, because inversion disrupts the global face-like configuration. Furthermore, the perception of a face in these stimuli is independent of the identification of the local parts. For instance, Moscovitch *et al.*^13^ reported the case of a visual agnostic patient who was impaired at identifying the local elements in Arcimboldo images (e.g. fruits, vegetables) though being still able to perceive the face. From a developmental point of view, the ability to perceive faces in these stimuli emerges early in the course of development. Kobayashi *et al.*^14^ found that 7–8 month-olds infants were able to perceive faces in Arcimboldo images. In addition, electrophysiological and functional neuroimaging studies have shown that the perception of pareidolic faces is hardwired in the human brain and is accompanied by typical face-related cortical activity (e.g.^9^,^12^,^15^,^16^,^17^). For instance, Hadjikhani *et al.*^15^ found that face-like objects (FLOs) evoked an early signal at 170 ms in the ventral fusiform cortex at a time and location similar to that evoked by real faces. Further evidence comes from an event-related fMRI study using Mooney and Arcimboldo images^9^: in that study, the categorization of a Mooney or Arcimboldo image as a face activated primarily the right fusiform faces area (FFA). Taken together, these results suggest that the ability to perceive faces in non-face stimuli cannot be attributed to a later cognitive reinterpretation process of the global face-like configuration.

The fact that non-face stimuli can be as easily perceived as faces solely on the basis of a global face-like configuration has been regarded as the most compelling evidence for a holistic/configural view of face perception^9^,^10^,^11^,^12^. According to this view, faces are processed as integrated wholes (referred to hereafter as ‘holistic/configural processing’) rather than as a collection of independent parts^18^,^19^. Two levels of holistic/configural processing can be distinguished^9^,^20^: one that allows the categorization of a given stimulus as a face based on a basic, coarse, representation of a face and a second stage that allows face individualization based on a more fine grained representation of the whole face. In the present study, we focus specifically on the former one.

In populations known to display deficits in face perception, the use of pareidolic faces may help to further understand the nature of their impairments with faces^9^,^13^,^20^. More specifically for individuals with ASD, the use of pareidolic faces further provides the advantage of studying face perception without being confounded by social factors necessarily present in real faces, notably the eyes, which have been showed to elicit an increased physiological response (e.g.^21^,^22^,^23^,^24^).

ASD is a neurodevelopmental condition marked by persistent deficits in socio-communicative domains together with the presence of restricted and repetitive patterns of behavior, interests or activities^25^. The study of face perception in this population is theoretically grounded by behavioral evidence of diminished orientation towards faces, impaired eye contact, as well as by a range of difficulties in face recognition and discrimination tasks (e.g.^26^,^27^). However, although impairments with faces are well documented in ASD, the nature of these impairments is still poorly understood and remains a topic of debate^26^,^27^,^28^,^29^,^30^,^31^,^32^.

According to one view, deficit in face perception in ASD stem from a perceptual origin^32^,^33^,^34^. This perceptual account posits that individuals with ASD process faces using a different strategy, based on an independent analysis of face parts. Whereas typically developing individuals (TD) process faces holistically, individuals with ASD would preferentially process the local facial features rather than the global configuration of faces. Support for this view comes from early findings showing better performance at recognizing isolated facial features and inverted faces^35^,^36^, as well as superior processing of face parts^37^. Studies manipulating spatial frequencies have also been taken as evidence for a locally oriented analysis of faces in ASD. Several of these studies reported a bias towards high spatial frequencies, which are thought to convey local rather than global information^38^,^39^. Importantly, this perceptual account of face perception deficit in ASD does not postulate a deficit of holistic/configural processing of faces in ASD. Rather, it is assumed that individuals with ASD are biased towards a part-based analysis^33^,^34^. As a corollary, it is expected that individuals with ASD outperformed TD individuals in tasks where it is advantageous to rely on local information. However, in tasks involving a global representation, individuals with ASD are thought to display inferior performance. According to this view, we might therefore postulate that young children with ASD would be impaired at perceiving faces in non-face stimuli on the basis of a global face-like configuration.

However, this analytical view of face perception in ASD has been challenged by many. In their literature review, Weigelt *et al.*^26^ noted that in the majority of studies, individuals with ASD are found to be sensitive to face manipulations known to probe holistic/configural processing. They noted that 12 out of 14 studies that manipulated face orientation evidenced a significant face inversion effect in individuals with ASD (e.g.^37^,^40^). Further evidence comes from studies demonstrating the presence of the composite face^41^ and part-whole^42^,^43^,^44^ effect in ASD, as well as intact sensitivity to the Thatcher illusion^24^,^45^,^46^. Moreover, several fMRI studies of face perception in ASD found normal FFA activation when gaze patterns and attention to faces, and notably to the eyes, were controlled^24^,^47^,^48^,^49^. Rather than being perceptual in nature, some have proposed that face-related impairments in ASD would primarily stem from an eye-avoidance strategy resulting from the eye region of the face being perceived as socially threatening^27^. Diminished eye fixation is among the most frequently reported finding in eye-tracking studies^50^,^51^,^52^ and electrodermal studies found greater skin conductance in response to direct eye contact in ASD^21^,^22^. Furthermore, several fMRI studies reported hyperactivation of the subcortical face processing pathway when individuals with ASD were cued to look into the eyes^24^ or were presented fearful faces gazing at them^23^. Dalton *et al.*^34^ have also demonstrated a positive correlation between amygdala activation and time spent in the eyes region in individuals with ASD. According to this last view, if indeed, individuals with ASD process face holistically, we might therefore expect intact perception of faces in non face-stimuli on the basis of a face-like configuration.

Whether individuals with ASD perceive faces in non-face stimuli has recently been investigated in high functioning adolescents^53^. In a first experiment, participants were asked to rate the face-likeness of objects on a Likert scale. The ratings of adolescents with ASD were similar to those of TD adolescents: objects that were rated as highly face-like were the same across groups. In a second experiment, the authors studied ERP responses to face-like objects (FLOs) and non-FLOs and found a face-likeness effect on the N170 amplitudes in both groups. Altogether, these results suggest that high functioning adolescents with ASD are as sensitive to pareidolic faces as TD individuals.

In the present study, we explored the perception of pareidolic faces in preschoolers with ASD using a preferential looking task to investigate orientation and attention maintenance towards upright FLOs. We presented upright FLOs and their inverted (upside down) version side by side. Because inversion disrupts the global face-like configuration, inverted FLOs are usually not perceived as face-like. Initial visual orientation was measured as the direction of the first fixation. We hypothesized that if the global face-like configuration had an effect on initial visual orientation, then children would be more likely to direct their first fixation towards upright rather than inverted FLOs. Attention maintenance was measured as the time spent looking at upright vs. inverted FLOs. We hypothesized that if children were sensitive to the global face-like configuration of upright FLOs, they would look longer at upright vs. inverted FLOs. Conversely, if children were not sensitive to the face-likeness of upright FLOs, we predicted that FLOs orientation would have no effect on looking time.

## Results

General and generalized mixed-effects models were used to reveal the main effect of FLOs orientation, group and their interaction; verbal and non-verbal mental ages were taken into consideration in every step. In terms of total fixation time, the mixed-effects model revealed a significant effect of FLOs orientation, indicating that total fixation time for upright stimuli were significantly longer, z = 2.02, *p* < .05, β = 0.19 (Table 1). This effect is observed in both groups as the effects of group, and their interactions were non-significant. To further characterize looking time between upright and inverted FLOs for each group, thereby probing the effect of face-likeness in maintaining attention at the early stage of processing, we computed the estimated mean first visit duration of each FLO (i.e. the amount of looking time on FLOs, the first time they are looked at). The mixed-effects model revealed a significant effect of FLOs orientation, indicating that first visit duration for upright FLOs were significantly longer, z = 1.98, *p* < .05, β = 0.14 (Table 1). Again, this effect is observed in both groups as the effects of group, and their interactions were non-significant.

**Figure 1:**
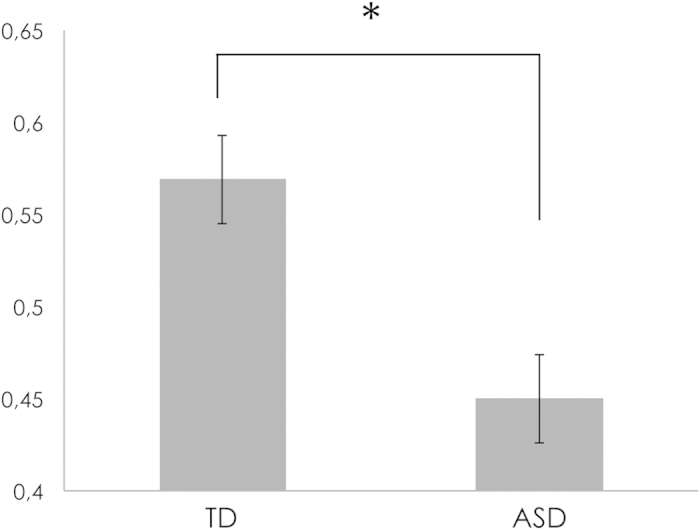
Probability of directing the first fixation towards upright FLOs in TD and ASD, *p < .05.

In terms of the probability of directing the first fixation towards upright FLOs, generalized mixed-effects model revealed a significant main effect of group indicating that the probability of looking first towards upright FLOs is significantly higher in the TD group (estimated probability = 0.57, log it = 0.28, *SE* = 0.10), compared to the ASD group (estimated probability = 0.45, log it = −0.22, *SE* = 0.12), z = 2.43, *p* < .05, OR = 1.59 (Fig. 1).

To investigate the robustness of our results, given the discrepancy between the numbers of participants in each group (resulting from the exclusion of four participants with ASD, see Methods), we reanalyzed our data, removing six TD children, in the way that the variability in the control group is maximized (so the probability of a type I error is minimized). This had no effect on the level of significance for any effects. Generalized mixed-effects model revealed a significant main effect of group on the probability of directing the first fixation towards upright FLOs, z = 2.35, *p* < .05, OR = 1.68. Mixed-effects models revealed a significant effect of FLOs orientation for the total fixation time, z = 2.91, *p* < .01, β = 0.23, and for the first visit duration, z = 2.07, *p* < .05, β = 0.16.

## Discussion

In the current study we asked whether young children with ASD were able to orient to and perceive a face in non-face stimuli on the basis of a global face-like configuration. First, we studied orientation towards upright FLOs measuring the direction of the first fixation. We found a behavioral dissociation between the two groups, showing that TD children were more likely to direct their first fixation towards upright FLOs than young children with ASD. Second, we measured looking times on upright and inverted FLOs and found that both groups looked significantly longer at upright vs. inverted FLOs. This result was found for the total looking time and the first visit duration. We will discuss these findings and their implications in turn.

In a context where faces are not presented centrally, they need to be (1) detected parafoveally, and (2) oriented towards in order for normal face processing to occur^54^. This ‘parafoveal detection’ is thought to be mainly subserved by a rapid and automatic subcortical neural pathway, including the superior colliculus, the pulvinar complex, and the amygdala^4^,^5^. In the first months of life, the detection of faces would automatically trigger preferential orienting towards them at a time when it is difficult to infants to select relevant information in their environment, thereby ensuring the proper development of specialized face processing and social brain networks^5^,^55^. However, with the development of attention control, these two different, yet closely linked, processes would dissociate. It is important to note that although the existence of a subcortical pathway has been disputed^56^, there is mounting evidence in the recent years to support the existence of this pathway both at the anatomical and functional level^5^,^57^.

Our analysis of the direction of the first fixation revealed that children with ASD were less likely than controls to detect and orient first towards upright FLOs. This result somewhat echoes findings of diminished orientation towards faces in ASD emerging at around 12 months of age (e.g.^58^,^59^). Some have proposed that a poor orientation towards faces may be caused by an innate deficit of the subcortical pathway^4^. However, a primary deficit of this neural pathway in ASD has recently been questioned^60^. Prospective studies of infants at high-risk for ASD indicate that within the first six months of life those who later develop ASD show typical spontaneous orienting and engagement with faces and eyes^59^,^60^,^61^,^62^. Notably, Jones and Klin^62^ established that at 2 months, infants at high-risk later diagnosed with ASD looked more at the eyes than TD infants. Our results showing reduced orientation may reflect a delay in maturation of the face processing network in ASD (e.g.^63^), as both Akechi *et al.*^64^ and Shah *et al.*^65^ showed that in older individuals with ASD there was a preferential detection of simple face-like stimuli (protoface). Note however that these studies investigated conscious awareness and covert orienting respectively while we studied overt orienting in the present study. Differences in outcome measures could also be a reason for the discrepancies in findings. Furthermore, as stated in the introduction, there is also evidence for an abnormal hyperactivation of the subcortical pathway in ASD when participants are cued to look into the eyes^24^ or are presented fearful faces gazing at them^23^. In contrast, other studies have reported that stimuli known to engage subcortical processes, averted eyes in a fearful face^66^ and fearful bodies^67^ , failed to activate the subcortical pathway in ASD^24^,^68^. Altogether these results may point to an abnormal tuning of the subcortical pathway in ASD, which under certain circumstances may lead to typical responses (e.g. Akechi *et al.*^64^, Shah *et al.*^65^). We thus agree with the view that a primary deficit of this neural pathway is unlikely in ASD. We nevertheless postulate that an abnormal tuning of the subcortical pathway in ASD may strongly interfere with orientation while leaving detection rather spared. Interestingly, Johnson *et al.*^5^ also discussed the possibility that eye contact processing and face processing in the subcortical pathway may be distinct processes. Further studies need to address the integrity of the subcortical face-processing pathway in ASD and its neurodevelopmental aspect as well as how and to what extent it interacts with attention control processes.

Our results also revealed a preference for upright FLOs in both groups. This indicates that young children with ASD, like TD controls, were sensitive to the global face-like configuration of upright FLOs. This finding extends the previous report of intact FLOs perception in high functioning adolescents with ASD^53^ in two ways. First, we demonstrate that even young children with ASD likely perceive a face in non-face objects on the basis of a global face-like configuration. Second, we also demonstrate that this face perception occurs spontaneously in ASD, without being explicitly cued, further confirming the view that perceiving faces in non-face stimuli relies on an early integrative process of the global face-like configuration. These findings therefore add to the evidence of holistic/configural processing of faces in ASD^26^.

According to the perceptual hypothesis of face perception in ASD, it was expected that FLOs orientation would have no effect on looking time in young children with ASD. This was not the case, further confirming that a bias towards local elements is unlikely to be the leading cause of abnormal face perception in ASD^26^,^27^. Rather, we agree with the view that impairments with faces in ASD primarily stem from an eye avoidance strategy resulting from an increased physiological response to the eyes^27^. Although this strategy can be regarded as adaptive (in the sense that it would decrease the overall level of arousal), we would like to argue that, without preventing holistic/configural processing of faces, this strategy nevertheless interferes with fine-grained holistic processing of individual faces. More specifically, it is likely that this eye-avoidance strategy lead to a difficulty in integrating simultaneously the multiple facial features provided by this region into a single representation, resulting in a reduced refined representation of this specific region. This view is in agreement with the evidence of impaired fine-grained discrimination of eye gaze direction^69^ as well as lack of discrimination of subtle changes in spacing affecting the eyes but not the mouth^43^,^70^.

Perceiving faces in non-face stimuli has been shown to rely primarily on the right hemisphere^9^,^10^. This is in agreement with the evidence of right hemispheric dominance for face perception (e.g.^71^,^72^,^73^,^74^). This right hemispheric dominance is usually explained by a differential involvement of each hemisphere in face processing^75^,^76^,^77^: while the left hemisphere is thought to be mostly involved in part-based processing, the right hemisphere is regarded as being mostly involved in holistic/configural processing. Although studies on hemispheric lateralization for face perception are scarce in ASD, there are a number of evidence that indicate a lack of right hemispheric dominance in processing faces among individuals with ASD. For instance, McPartland *et al.*^78^, studying ERPs, found a reduced right lateralization effect for faces in ASD compared to TD individuals. In another study, Ashwin *et al.*^79^ found a reduced bias to the left visual field in a chimeric face task. In addition, a lack of left gaze bias in ASD has also been found^80^,^81^. Studies of infants at high risk for ASD also reveal atypical right hemispheric specialization^82^,^83^,^84^. For now, these results are difficult to reconcile with a holistic/configural processing of face and intact perception of FLOs. However, it is important to keep in mind that the right hemispheric dominance for face perception reflects a relative dominance of one hemisphere over the other in early stages of holistic processing rather than absolute differences. Further investigations will be necessary to explore specifically the right hemisphere contribution to the perception of pareidolic faces in ASD and more broadly, to investigate hemispheric specialization as well as inter-hemispheric cooperation for face perception.

Pellicano and Burr^85^ have proposed that individuals with ASD may have attenuated Bayesian priors, leading to a different, more accurate perception of the world. The present data add to previous studies demonstrating that individuals with ASD are sensitive to pareidolic stimuli, and that for that category at least, they have similar perceptual inferences as TD controls, going against Pellicano and Burr’s assumption. There may however be a special status for social stimuli such as faces – that in one hand can be ‘overgeneralized’ as shown here with FLOs, but that on the other hand lead to abnormal orienting mechanisms, via an abnormal subcortical pathway linked to abnormal action control processes, maybe through alterations of the dopaminergic pathways that in turn reduce saliency of these social stimuli^86^.

In conclusion, using a preferential looking task, we found that young children with ASD were less likely to orient first towards upright FLOs than TD, showing that a global face-like configuration initially bias orientation of ASD children to a lesser degree than is observed in TD. However, once upright FLOs are foveated, both groups look longer at these stimuli, suggesting that they both perceive them as a face. We suggest that abnormal orientation to upright FLOs may stem from an abnormal tuning of the subcortical pathway that fails to increase attention and orientation towards biologically relevant stimuli. Once foveated, however, upright FLOs may elicit activation in the classical face-processing network, as suggested by increased looking time for upright vs. inverted stimuli. Additional investigations using pareidolic faces are needed. Notably, it will be necessary to further explore the neural bases of pareidolic face perception in individuals with ASD. The use of these pareidolic faces bears the potential of a better understanding of the integrity, both at a behavioral and neural level, of the face-processing routes in ASD.

## Methods

### Participants

Participants included 17 preschoolers with ASD aged 24–60 months and 23 age-matched TD peers (see Table 2). An additional four preschoolers with ASD participated in this study but had to be excluded from the analysis because of poor calibration due to fussiness. Children in the TD group had no history of social or cognitive developmental concerns and no family history of ASD in first- or second- degree relatives by parent report. The diagnosis of ASD was established using algorithm cut-offs on the ADOS (module 1 or 2) and the ADI-R and was further confirmed by one expert clinician (BR or JK) based on DSM-5 criteria and a review of all available information. To standardize ADOS scores between modules, we also computed a severity score based on Gotham *et al.*^87^. They had no known specific neurological disorders or genetic conditions.

Given reports regarding the prevalence of developmental delays in preschoolers with ASD (e.g. Chawarska *et al.*^88^), nonverbal and verbal developmental level were assessed in both groups using the Mullen Scales of Early Learning at the time children were included in the study.

An independent samples t-test was performed to test for between-group difference in chronological age, nonverbal and verbal developmental level. We used a chi-square test of independence to examine difference in sex ratio. The ASD group did not differ from the TD group in terms of chronological age, *t*(38) = −0.59, *p* > .05 and sex ratio, *χ2* (1, N = 40) = 2.15, *p* > .05. However, as expected, TD children had significantly higher nonverbal, *t*(38) = −2.41, *p* < .05, and verbal, *t*(38) = −3.17, *p* < .005, developmental level (see Table 2).

All parents gave their free and informed consent to the study before testing in accordance with the Declaration of Helsinki and all procedures were approved by the local Ethics Committee (CPP). All procedures were carried out in accordance with the approved protocol.

### Stimuli

Fourteen color photographs of FLOs, from the book “FACES” by François Robert and Jean Robert, were used for this study. They were selected from those used in our previous study and they all have been interpreted as a face by neurotypical adults in a face-like/no face-like categorization task (for grayscale examples of these stimuli see Fig. 1 from Hadjikhani *et al.*^15^). The width of the photographs was standardized to 400 pixels (10.1° at 60 cm). The heights were adjusted so as not to change original proportions. The resulting heights ranged from 354 to 520 pixels (9.0° to 13.2° respectively at 60 cm). The letters and numbers displayed in certain objects were digitally removed to prevent any attentional bias towards these categories, which are known to be centers of narrow interests in preschoolers with ASD^89^,^90^.

### Procedure

Stimuli were presented using E-prime 2.0 software (Psychology Software Tools Inc., Pittsburgh, PA) on a 17-inch screen (1280 × 1024 pixels), integrated to a Tobii T120 eye-tracker (Tobii Inc., Stockholm, Sweden). The pupil and corneal reflections were recorded binocularly at a sampling rate of 60 Hz. The recordings were made in room where light levels were kept constant for all participants. All potential distractors were removed, and children sat in a car seat adapted and fixed to a chair with adjustable height at a distance of about 60 cm from the screen. If they so desired, parents could stay with their children, provided they remained behind the seat and did not talk to the child during the experiment. Prior to the test, a 5-point calibration was performed. The calibration point was a video of 1.5° × 1.5° representing a bouncing ball accompanied by a sound, displayed successively at each of the four corners of the screen and its center. Calibration was repeated until the five calibration points had sufficient quality as judged by visual inspection of gaze plots provided by the eye tracker.

**Figure 2:**
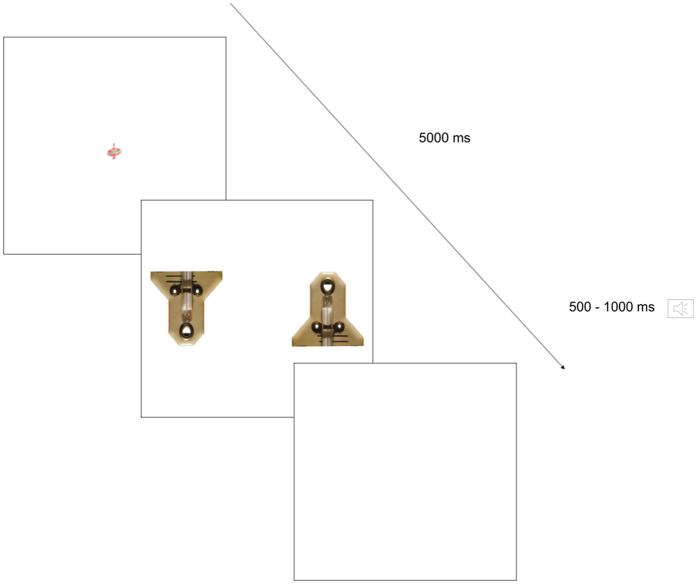
Description of a trial from the preferential-looking task. Photo by Francois & Jean ROBERT at icuc@mac.com

A preferential-looking task was used for this study (Fig. 2). The experimental session consisted of 14 trials where an upright and an inverted FLO were presented bilaterally on the left and right side of the screen. The two FLOs were identical except for their orientation. A distance of 8.1° separated the inner side of the two FLOs (i.e. about 4° away from the center of the screen). Upright and inverted FLOs were counterbalanced by side such that half the trials had upright FLOs on the left and half had them on the right. Each trial began with a 1.5° × 1.5° central fixation point representing a colorful spinning top on a white background. We used an active gaze contingent procedure to ensure that upright and inverted FLOs were equidistant from the participants’ view when they appeared on the screen. For the FLOs to appear, participants had to look at the spinning top for at least 300 consecutive milliseconds within a time window of 5 seconds. Otherwise, the stimuli were displayed after 5 seconds had elapsed, and the trial was not included in the analysis. Paired FLOs were presented for 5 seconds in random order, followed by a white screen accompanied by a sound varying in length from 500 to 1000 milliseconds to maintain the child’s attention on the task.

### Data analysis

Pre-processing of raw gaze data were done in Matlab based on algorithms developed by Wass *et al.*^91^. Fixations were identified using a velocity threshold of 35° s^−1^ and with a minimum duration threshold of 100 milliseconds. Trials were included in subsequent analyses only (1) if children were looking at the central fixation point when they shifted their gaze toward FLOs (upright or inverted) and (2) if a minimum fixation time of 2100 milliseconds (5^th^ percentile) on FLOs was available for analysis. This threshold was used to target trials with insufficient data resulting from either children not being engaged or low robustness (i.e. fragmented contact with the eye tracker). Children in the ASD group contributed an average of 10.5 (75%) trials (*SD* = 1.9) and children in the TD group contributed an average of 10.9 (78%) trials (*SD* = 2.4). An independent samples t-test revealed no significant difference between groups, *t*(38) = −0.54 , *p* > .05.

A crossed random effect mixed model was used to address our research questions. This statistical approach has several advantages such as 1) flexibility in addressing dependencies among observations, 2) possibility of simultaneous examination of the main effects and interactions of categorical and continuous variables, 3) flexibility in handling missing data coming from individuals with only partial response^92^,^93^. Considering these advantages this approach has become increasingly popular in eye-tracking studies. Mixed effect models provide the possibility of both testing the independent variables of interest and their interactions in terms of fixed-effects (in our case, group, FLOs orientation and developmental levels) as well as other dependencies in the data structure which may affect the outcome in terms of random-effects (in our case, individual participants and individual stimuli). All analyses were performed using R software, package lme4^94^.

## Additional Information

**How to cite this article**: Guillon, Q. *et al.* Intact perception but abnormal orientation towards face-like objects in young children with ASD. *Sci. Rep.* **6**, 22119; doi: 10.1038/srep22119 (2016).

## Figures and Tables

**Table 1:** Estimated means and standard errors for total fixation time and first visit duration for upright and inverted FLOs by group.

	ASD		TD	
	Upright	Inverted	Upright	Inverted
Total fixation time (ms)	2056 (75)	1774 (55)	2070 (71)	1879 (73)
First visit duration (ms)	1252 (93)	1110 (95)	1303 (74)	1170 (80)

**Table 2:** Sample characteristics.

	ASD (*n* = 17)		TD (*n* = 23)	
	M	SD	M	SD
CA	40.9	12.1	43.5	14.3
NVMA^*^	34.9	13.0	45.6	14.6
VMA^*^	29.5	13.8	44.3	15.2
Male/female	14/3		14/9	
ADOS severity score	6.6	1.6	N/A	

*ASD*, autism spectrum disorder; *TD*, typical development; *CA*, chronological age in months; *NVMA*, non-verbal mental age in months; *VMA*, verbal mental age in months.

*p < 0.05.

## References

[b1] Palermo R. & Rhodes G. Are you always on my mind? A review of how face perception and attention interact. Neuropsychologia 45, 75–92 (2007). 16797607 10.1016/j.neuropsychologia.2006.04.025

[b2] Evritt L. *Pareidolia: Why we see faces in hills, the Moon and toasties*. BBC News Magazine. (2013) Available at: http://www.bbc.com/news/magazine-22686500 (Accessed: 14th December 2015).

[b3] Verpooten J. & Nelissen M. Sensory exploitation and cultural transmission: The late emergence of iconic representations in human evolution. Theory Biosci. 129, 211–221 (2010). 20556543 10.1007/s12064-010-0095-7

[b4] Johnson M. H. Subcortical face processing. Nat. Rev. Neurosci. 6, 766–74 (2005). 16276354 10.1038/nrn1766

[b5] Johnson M. H., Senju A. & Tomalski P. The two-process theory of face processing: Modifications based on two decades of data from infants and adults. Neurosci. Biobehav. Rev. 50, 169–179 (2015). 25454353 10.1016/j.neubiorev.2014.10.009

[b6] Morton J. & Johnson M. H. CONSPEC and CONLERN: a two-process theory of infant face recognition. Psychol. Rev. 98, 164–81 (1991). 2047512 10.1037/0033-295x.98.2.164

[b7] Diamond R. & Carey S. Why faces are and are not special: an effect of expertise. J. Exp. Psychol. Gen. 115, 107–17 (1986). 2940312 10.1037//0096-3445.115.2.107

[b8] Mooney G. Age in the development of closure ability in children. Can. J. Psychol. 11, 219–226 (1957). 13489559 10.1037/h0083717

[b9] Rossion B., Dricot L., Goebel R. & Busigny T. Holistic face categorization in higher order visual areas of the normal and prosopagnosic brain: toward a non-hierarchical view of face perception. Front. Hum. Neurosci. 4, 225 (2011). 21267432 10.3389/fnhum.2010.00225 PMC3025660

[b10] Parkin A. J. & Williamson P. Cerebral Lateralisation at Different Stages of Facial Processing. Cortex 23, 99–110 (1987). 3568709 10.1016/s0010-9452(87)80022-9

[b11] McKone E. Isolating the special component of face recognition: peripheral identification and a Mooney face. J. Exp. Psychol. Learn. Mem. Cogn. 30, 181–97 (2004). 14736306 10.1037/0278-7393.30.1.181

[b12] Caharel S. *et al.* Early holistic face-like processing of Arcimboldo paintings in the right occipito-temporal cortex: evidence from the N170 ERP component. Int. J. Psychophysiol. 90, 157–64 (2013). 23816562 10.1016/j.ijpsycho.2013.06.024

[b13] Moscovitch M., Winocur G. & Behrmann M. What Is Special about Face Recognition? Nineteen Experiments on a Person with Visual Object Agnosia and Dyslexia but Normal Face Recognition. J. Cogn. Neurosci. 9, 555–604 (1997). 23965118 10.1162/jocn.1997.9.5.555

[b14] Kobayashi M. *et al.* Do infants recognize the Arcimboldo images as faces? Behavioral and near-infrared spectroscopic study. J. Exp. Child Psychol. 111, 22–36 (2012). 21875715 10.1016/j.jecp.2011.07.008

[b15] Hadjikhani N., Kveraga K., Naik P. & Ahlfors S. P. Early (M170) activation of face-specific cortex by face-like objects. Neuroreport 20, 403–7 (2009). 19218867 10.1097/WNR.0b013e328325a8e1 PMC2713437

[b16] Churches O., Baron-Cohen S. & Ring H. Seeing face-like objects: an event-related potential study. Neuroreport 20, 1290–4 (2009). 19641476 10.1097/WNR.0b013e3283305a65

[b17] Latinus M. & Taylor M. J. Holistic processing of faces: learning effects with Mooney faces. J. Cogn. Neurosci. 17, 1316–27 (2005). 16197686 10.1162/0898929055002490

[b18] Young A. W., Hellawell D. & Hay D. C. Configurational information in face perception. Perception 16, 747–759 (1987). 3454432 10.1068/p160747

[b19] Tanaka J. & Farah M. J. Parts and wholes in face recognition. Q. J. Exp. Psychol. A. 46, 225–45 (1993). 8316637 10.1080/14640749308401045

[b20] Busigny T., Joubert S., Felician O., Ceccaldi M. & Rossion B. Holistic perception of the individual face is specific and necessary: evidence from an extensive case study of acquired prosopagnosia. Neuropsychologia 48, 4057–92 (2010). 20875437 10.1016/j.neuropsychologia.2010.09.017

[b21] Kylliäinen A. & Hietanen J. K. Skin conductance responses to another person’s gaze in children with autism. J. Autism Dev. Disord. 36, 517–525 (2006). 16555137 10.1007/s10803-006-0091-4

[b22] Joseph R. M., Ehrman K., McNally R. & Keehn B. Affective response to eye contact and face recognition ability in children with ASD. J. Int. Neuropsychol. Soc. 14, 947–55 (2008). 18954475 10.1017/S1355617708081344

[b23] Zürcher N. R. *et al.* Perception of social cues of danger in autism spectrum disorders. PLoS One 8, e81206 (2013). 24324679 10.1371/journal.pone.0081206 PMC3852523

[b24] Zürcher N. R. *et al.* It’s all in the eyes: subcortical and cortical activation during grotesqueness perception in autism. PLoS One 8, e54313 (2013). 23342130 10.1371/journal.pone.0054313 PMC3544832

[b25] American Psychiatric Association. Diagnostic and Statistical Manual of Mental Disorders (Fifth Edition). (American Psychiatric Association, 2013).

[b26] Weigelt S., Koldewyn K. & Kanwisher N. Face identity recognition in autism spectrum disorders: a review of behavioral studies. Neurosci. Biobehav. Rev. 36, 1060–84 (2012). 22212588 10.1016/j.neubiorev.2011.12.008

[b27] Tanaka J. & Sung A. The ‘Eye Avoidance’ Hypothesis of Autism Face Processing. J. Autism Dev. Disord. doi: 10.1007/s10803-013-1976–7 (2013). PMC3997654 24150885

[b28] Schultz R. T. Developmental deficits in social perception in autism: the role of the amygdala and fusiform face area. Int. J. Dev. Neurosci. 23, 125–41 (2005). 15749240 10.1016/j.ijdevneu.2004.12.012

[b29] Sasson N. The development of face processing in autism. J. Autism Dev. Disord. 36, 381–94 (2006). 16572261 10.1007/s10803-006-0076-3

[b30] Jemel B., Mottron L. & Dawson M. Impaired face processing in autism: fact or artifact? J. Autism Dev. Disord. 36, 91–106 (2006). 16477517 10.1007/s10803-005-0050-5

[b31] Tang J. *et al.* Face Recognition and Visual Search Strategies in Autism Spectrum Disorders: Amending and Extending a Recent Review by Weigelt *et al*. PLoS One 10, e0134439 (2015). 26252877 10.1371/journal.pone.0134439 PMC4529109

[b32] Behrmann M., Thomas C. & Humphreys K. Seeing it differently: visual processing in autism. Trends Cogn. Sci. 10, 258–264 (2006). 16713326 10.1016/j.tics.2006.05.001

[b33] Mottron L., Dawson M., Soulières I., Hubert B. & Burack J. Enhanced perceptual functioning in autism: an update, and eight principles of autistic perception. J. Autism Dev. Disord. 36, 27–43 (2006). 16453071 10.1007/s10803-005-0040-7

[b34] Happé F. & Frith U. The weak coherence account: detail-focused cognitive style in autism spectrum disorders. J. Autism Dev. Disord. 36, 5–25 (2006). 16450045 10.1007/s10803-005-0039-0

[b35] Hobson R. P., Ouston J. & Lee A. What’s in a face? The case of autism. Br. J. Psychol. 79, 441–453 (1988). 3208000 10.1111/j.2044-8295.1988.tb02745.x

[b36] Langdell T. Recognition of faces: an approach to the study of autism. J. Child Psychol. Psychiatry 19, 255–268 (1978). 681468 10.1111/j.1469-7610.1978.tb00468.x

[b37] Lahaie a. *et al.* Face perception in high-functioning autistic adults: evidence for superior processing of face parts, not for a configural face-processing deficit. Neuropsychology 20, 30–41 (2006). 16460220 10.1037/0894-4105.20.1.30

[b38] Deruelle C., Rondan C., Gepner B. & Tardif C. Spatial frequency and face processing in children with autism and Asperger syndrome. J. Autism Dev. Disord. 34, 199–210 (2004). 15162938 10.1023/b:jadd.0000022610.09668.4c

[b39] Deruelle C., Rondan C., Salle-Collemiche X., Bastard-Rosset D. & Da Fonséca D. Attention to low- and high-spatial frequencies in categorizing facial identities, emotions and gender in children with autism. Brain Cogn. 66, 115–23 (2008). 17693004 10.1016/j.bandc.2007.06.001

[b40] Scherf K. S., Behrmann M., Minshew N. & Luna B. Atypical development of face and greeble recognition in autism. J. Child Psychol. Psychiatry 49, 838–47 (2008). 18422548 10.1111/j.1469-7610.2008.01903.x PMC3071970

[b41] Nishimura M., Rutherford M. D. & Maurer D. Converging evidence of configural processing of faces in high-functioning adults with autism spectrum disorders. Vis. cogn. 16, 859–891 (2008).

[b42] Joseph R. M. & Tanaka J. Holistic and part-based face recognition in children with autism. J. Child Psychol. Psychiatry 44, 529–42 (2003). 12751845 10.1111/1469-7610.00142

[b43] Wolf J. *et al.* Specific impairment of face-processing abilities in children with autism spectrum disorder using the Let’s Face It! skills battery. Autism Res. 1, 329–40 (2008). 19360688 10.1002/aur.56 PMC4589218

[b44] Faja S., Webb S. J., Merkle K., Aylward E. & Dawson G. Brief report: face configuration accuracy and processing speed among adults with high-functioning autism spectrum disorders. J. Autism Dev. Disord. 39, 532–8 (2009). 18751880 10.1007/s10803-008-0635-x PMC2977974

[b45] Rouse H., Donnelly N., Hadwin J. & Brown T. Do children with autism perceive second-order relational features? The case of the Thatcher illusion. J. Child Psychol. Psychiatry 45, 1246–57 (2004). 15335345 10.1111/j.1469-7610.2004.00317.x

[b46] Cleary L., Brady N., Fitzgerald M. & Gallagher L. Holistic processing of faces as measured by the Thatcher illusion is intact in autism spectrum disorders. Autism doi: 10.1177/1362361314526005 (2014). 24637429

[b47] Hadjikhani N. *et al.* Activation of the fusiform gyrus when individuals with autism spectrum disorder view faces. Neuroimage 22, 1141–50 (2004). 15219586 10.1016/j.neuroimage.2004.03.025

[b48] Dalton K. M. *et al.* Gaze fixation and the neural circuitry of face processing in autism. Nat. Neurosci. 8, 519–26 (2005). 15750588 10.1038/nn1421 PMC4337787

[b49] Perlman S. B., Hudac C. M., Pegors T., Minshew N. & Pelphrey K. a. Experimental manipulation of face-evoked activity in the fusiform gyrus of individuals with autism. Soc. Neurosci. 6, 22–30 (2011). 20446172 10.1080/17470911003683185 PMC3093050

[b50] Falck-Ytter T. & von Hofsten C. How special is social looking in ASD: a review. Prog. Brain Res. 189, 209–22 (2011). 21489391 10.1016/B978-0-444-53884-0.00026-9

[b51] Guillon Q., Hadjikhani N., Baduel S. & Rogé B. Visual social attention in autism spectrum disorder: Insights from eye tracking studies. Neurosci. Biobehav. Rev. 42C, 279–297 (2014). 24694721 10.1016/j.neubiorev.2014.03.013

[b52] Papagiannopoulou E. A., Chitty K. M., Hermens D. F., Hickie I. B. & Lagopoulos J. A systematic review and meta-analysis of eye-tracking studies in children with autism spectrum disorders. Soc. Neurosci. 1–23 doi: 10.1080/17470919.2014.934966 (2014). 24988218

[b53] Akechi H., Kikuchi Y., Tojo Y., Osanai H. & Hasegawa T. Neural and behavioural responses to face-likeness of objects in adolescents with autism spectrum disorder. Sci. Rep. 4, 3874 (2014). 24464152 10.1038/srep03874 PMC5379204

[b54] Lewis M. B. & Edmonds A. J. Face detection: Mapping human performance. Perception 32, 903–920 (2003). 14580138 10.1068/p5007

[b55] Grice S. J. *et al.* Neural Correlates of Eye-Gaze Detection in Young Children with Autism. Cortex 41, 342–353 (2005). 15871599 10.1016/s0010-9452(08)70271-5

[b56] Pessoa L. & Adolphs R. Emotion processing and the amygdala: from a ‘low road’ to ‘many roads’ of evaluating biological significance. Nat. Rev. Neurosci. 11, 773–783 (2010). 20959860 10.1038/nrn2920 PMC3025529

[b57] Tamietto M. & de Gelder B. Neural bases of the non-conscious perception of emotional signals. Nat. Rev. Neurosci. 11, 697–709 (2010). 20811475 10.1038/nrn2889

[b58] Osterling J. & Dawson G. Early recognition of children with autism: a study of first birthday home videotapes. J. Autism Dev. Disord. 24, 247–57 (1994). 8050980 10.1007/BF02172225

[b59] Ozonoff S. *et al.* A Prospective Study of the Emergence of Early Behavioral Signs of Autism. J. Am. Acad. Child Adolesc. Psychiatry 49, 256–266. e2 (2010). 20410715 PMC2923050

[b60] Johnson M. H. Autism: demise of the innate social orienting hypothesis. Curr. Biol. 24, R30–1 (2014). 24405675 10.1016/j.cub.2013.11.021

[b61] Elsabbagh M. *et al.* The development of face orienting mechanisms in infants at-risk for autism. Behav. Brain Res. 251, 147–54 (2013). 22846849 10.1016/j.bbr.2012.07.030 PMC3730054

[b62] Jones W. & Klin A. Attention to eyes is present but in decline in 2-6-month-old infants later diagnosed with autism. Nature 504, 427–31 (2013). 24196715 10.1038/nature12715 PMC4035120

[b63] Bakhtiari R. *et al.* Differences in white matter reflect atypical developmental trajectory in autism: A Tract-based Spatial Statistics study. NeuroImage Clin. 1, 48–56 (2012). 24179736 10.1016/j.nicl.2012.09.001 PMC3757732

[b64] Akechi H. *et al.* Preferential awareness of protofacial stimuli in autism. Cognition 143, 129–134 (2015). 26143377 10.1016/j.cognition.2015.06.016

[b65] Shah P., Gaule A., Bird G. & Cook R. Robust orienting to protofacial stimuli in autism. Curr. Biol. 23, R1087–8 (2013). 24355781 10.1016/j.cub.2013.10.034 PMC3898081

[b66] Hadjikhani N., Hoge R., Snyder J. & de Gelder B. Pointing with the eyes: The role of gaze in communicating danger. Brain Cogn. 68, 1–8 (2008). 18586370 10.1016/j.bandc.2008.01.008 PMC2582139

[b67] Hadjikhani N. & De Gelder B. Seeing Fearful Body Expressions Activates the Fusiform Cortex and Amygdala. Curr. Biol. 13, 2201–2205 (2003). 14680638 10.1016/j.cub.2003.11.049

[b68] Hadjikhani N. *et al.* Body expressions of emotion do not trigger fear contagion in autism spectrum disorder. Soc. Cogn. Affect. Neurosci. 4, 70–78 (2009). 19151375 10.1093/scan/nsn038 PMC2656879

[b69] Campbell R. *et al.* Meanings in motion and faces: Developmental associations between the processing of intention from geometrical animations and gaze detection accuracy. Dev. Psychopathol. 18, (2006). 10.1017/S0954579406060068 16478554

[b70] Rutherford M. D., Clements K. a. & Sekuler A. B. Differences in discrimination of eye and mouth displacement in autism spectrum disorders. Vision Res. 47, 2099–110 (2007). 17559905 10.1016/j.visres.2007.01.029

[b71] Hillger L. A. & Koenig O. Separable mechanisms in face processing: evidence from hemispheric specialization. J. Cogn. Neurosci. 3, 42–58 (1991). 23964804 10.1162/jocn.1991.3.1.42

[b72] Kanwisher N., McDermott J. & Chun M. M. The fusiform face area: a module in human extrastriate cortex specialized for face perception. J. Neurosci. 17, 4302–11 (1997). 9151747 10.1523/JNEUROSCI.17-11-04302.1997 PMC6573547

[b73] Bentin S., Allison T., Puce A., Perez E. & McCarthy G. Electrophysiological Studies of Face Perception in Humans. J. Cogn. Neurosci. 8, 551–565 (1996). 20740065 10.1162/jocn.1996.8.6.551 PMC2927138

[b74] Haxby J., Hoffman E. & Gobbini M. The distributed human neural system for face perception. Trends Cogn. Sci. 4, 223–233 (2000). 10827445 10.1016/s1364-6613(00)01482-0

[b75] Bourne V., Vladeanu M. & Hole G. J. Lateralised repetition priming for featurally and configurally manipulated familiar faces: evidence for differentially lateralised processing mechanisms. Laterality 14, 287–99 (2009). 18949655 10.1080/13576500802383709

[b76] Rossion B. *et al.* Hemispheric asymmetries for whole-based and part-based face processing in the human fusiform gyrus. J. Cogn. Neurosci. 12, 793–802 (2000). 11054921 10.1162/089892900562606

[b77] Ramon M. & Rossion B. Hemisphere-dependent holistic processing of familiar faces. Brain Cogn. 78, 7–13 (2012). 22099150 10.1016/j.bandc.2011.10.009

[b78] McPartland J. *et al.* Atypical neural specialization for social percepts in autism spectrum disorder. Soc. Neurosci. 6, 436–51 (2011). 21777159 10.1080/17470919.2011.586880 PMC3204335

[b79] Ashwin C., Wheelwright S. & Baron-Cohen S. Laterality Biases to Chimeric Faces in Asperger Syndrome: What is Right About Face-Processing? J. Autism Dev. Disord. 35, 183–196 (2005). 15909405 10.1007/s10803-004-1997-3

[b80] Dundas E., Best C. a., Minshew N. & Strauss M. S. A lack of left visual field bias when individuals with autism process faces. J. Autism Dev. Disord. 42, 1104–11 (2012). 21986874 10.1007/s10803-011-1354-2 PMC3428133

[b81] Guillon Q. *et al.* Both dog and human faces are explored abnormally by young children with autism spectrum disorders. Neuroreport 25, 1237–41 (2014). 25162783 10.1097/WNR.0000000000000257

[b82] McCleery J. P., Akshoomoff N., Dobkins K. R. & Carver L. Atypical face versus object processing and hemispheric asymmetries in 10-month-old infants at risk for autism. Biol. Psychiatry 66, 950–7 (2009). 19765688 10.1016/j.biopsych.2009.07.031 PMC2783702

[b83] Dundas E., Gastgeb H. Z. & Strauss M. S. Left visual field biases when infants process faces: a comparison of infants at high- and low-risk for autism spectrum disorder. J. Autism Dev. Disord. 42, 2659–68 (2012). 22527700 10.1007/s10803-012-1523-y PMC3408549

[b84] Keehn B., Vogel-Farley V., Tager-Flusberg H. & Nelson C. a. Atypical Hemispheric Specialization for Faces in Infants at Risk for Autism Spectrum Disorder. Autism Res. 1–12 doi: 10.1002/aur.1438 (2015). 25808162 PMC4412772

[b85] Pellicano E. & Burr D. When the world becomes ‘too real’: A Bayesian explanation of autistic perception. Trends Cogn. Sci. 16, 504–510 (2012). 22959875 10.1016/j.tics.2012.08.009

[b86] Chmielewski W. X. & Beste C. Action control processes in autism spectrum disorder – Insights from a neurobiological and neuroanatomical perspective. Prog. Neurobiol. 124, 49–83 (2015). 25450950 10.1016/j.pneurobio.2014.11.002

[b87] Gotham K., Pickles A. & Lord C. Standardizing ADOS scores for a measure of severity in autism spectrum disorders. J. Autism Dev. Disord. 39, 693–705 (2009). 19082876 10.1007/s10803-008-0674-3 PMC2922918

[b88] Chawarska K., Klin A., Paul R., Macari S. & Volkmar F. A prospective study of toddlers with ASD: short-term diagnostic and cognitive outcomes. J. Child Psychol. Psychiatry 50, 1235–45 (2009). 19594835 10.1111/j.1469-7610.2009.02101.x PMC4878113

[b89] Klin A., Danovitch J. H., Merz A. B. & Volkmar F. Circumscribed Interests in Higher Functioning Individuals With Autism Spectrum Disorders: An Exploratory Study. Res. Pract. Pers. with Sev. Disabil. 32, 89–100 (2007).

[b90] Sasson N. & Touchstone E. W. Visual attention to competing social and object images by preschool children with autism spectrum disorder. J. Autism Dev. Disord. 44, 584–92 (2014). 23918441 10.1007/s10803-013-1910-z

[b91] Wass S., Smith T. J. & Johnson M. H. Parsing eye-tracking data of variable quality to provide accurate fixation duration estimates in infants and adults. Behav. Res. Methods 45, 229–50 (2013). 22956360 10.3758/s13428-012-0245-6 PMC3578727

[b92] Baayen R. H., Davidson D. J. & Bates D. M. Mixed-effects modeling with crossed random effects for subjects and items. J. Mem. Lang. 59, 390–412 (2008).

[b93] Hoffman L. & Rovine M. J. Multilevel models for the experimental psychologist: foundations and illustrative examples. Behav. Res. Methods 39, 101–117 (2007). 17552476 10.3758/bf03192848

[b94] Bates D., Mächler M., Bolker B. & Walker S. Fitting Linear Mixed-Effects Models Using lme4. J. Stat. Softw. 67, (2015).

